# Knowledge and Attitudes Regarding Clinical Trial Participation: A Cross-Sectional Study in the Eastern Province, Saudi Arabia

**DOI:** 10.7759/cureus.47823

**Published:** 2023-10-27

**Authors:** Abdullah Almaqhawi, Arwa K AlHussain, Meshal Alharbi, Rawan S Alsaad, Mohammed K Alsuhayyih, Turki Alhumam, Mohammed Alamer, Abdulkarim H Almohammed, Mubarak M Bu Haya

**Affiliations:** 1 Family and Community Medicine, King Faisal University, Al-Ahsa, SAU; 2 College of Medicine, King Faisal University, Al-Ahsa, SAU; 3 Department of Basic Science, College of Medicine, Almareefa University, Riyadh, SAU

**Keywords:** clinical trail, public opinion, perception, attitude, knowledge

## Abstract

Objective

This study aims to assess the knowledge and attitudes toward clinical trial (CT) participation among the adult population in the Eastern Province of Saudi Arabia.

Material and methods

This cross-sectional study was conducted among the population of the Eastern Province of Saudi Arabia. A self-administered questionnaire was distributed among the general population using an online survey.

Results

A total of 334 participants completed the questionnaire. Participants' ages ranged from 18 to 65 years, with a mean age of 31.2 ± 13.9 years, 56.6% of whom were males, 42.2% were employed, 29.6% were students, and 23.1% were unemployed. Surprisingly, only a small percentage of respondents (7.5%) were requested to participate in a randomized controlled trial (RCT), of which the majority did partake. Additionally, 25.4% of participants believe CTs are used to evaluate new drugs; others believe that CTs are used to understand diseases and human behavior. The data show that most participants believe that CTs improve patient care, welfare, and society. Also, participants were more likely to take part if they were aware of the study's purpose and findings and were given more time to consider their options.

Conclusion

Participants believed that the biggest obstacle was a lack of knowledge of CTs. It is crucial to educate patients more about CTs. Multimodal strategies such as improved patient-provider communication and online information for trial information sharing may be effective in boosting knowledge and CT recruitment.

## Introduction

A clinical trial (CT) is a superior research technique for increasing medical knowledge and practice because it is regarded as the highest degree of evidence for medical practice and decision-making [1]. In addition to the individual and scientific advantages of CTs, they are critical in the creation and determination of the efficacy of novel medications and in improving patient care [2]. CTs are also the quickest and safest approach to finding out which therapy tactics and diagnostic tests work best [3]. The best available evidence for creating better treatments or interventions to treat difficult conditions and enhance the quality of life is found in observational studies or randomized controlled trials (RCTs). The ultimate objective of such trials and studies is to guarantee the highest possible standard of patient care with the greatest possible result while minimizing the expense and strain on the patient [4].

However, a few primary barriers to patients participating in clinical trials should be noted, including a lack of information and awareness regarding CTs. Fear, misunderstandings, perceptions, and beliefs of the patient are important barriers. Thus, the role of the physician in patient engagement in CTs is important because participants would be more comfortable participating in a CT if they perceived their doctors to be knowledgeable and trustworthy [5]. Currently, there are 415,701 registered CTs worldwide. North America has the highest number of CT studies in the world, with 170,214, of which 152,929 are being conducted in the United States alone. In the Middle East, there are 20,319 studies. In the Gulf region, Saudi Arabia is dominating with 1,023 studies in CTs, in contrast to the UAE (n = 318), Qatar (n = 174), Kuwait (n = 150), and Oman (n = 61) [6]. Many nations' healthcare systems have been severely harmed as a result of the public health measures brought about by the COVID-19 pandemic. Clinical trial participants, caregivers, researchers, trial sponsors, and research institutions were all significantly affected by this circumstance [4].

Despite the elevated level of safety offered to entice volunteers for CTs, recruiting has always been difficult, and most people are ignorant of CTs and how their participation helps create future treatments and technology [7]. Little empirical research on people's knowledge, attitudes, and views concerning CTs in developing nations and the Middle East exists [8,9]. However, the findings of a cross-sectional study with 232 Saudi adult patients and their companions visiting adult outpatient clinics at King Fahad Medical City disclosed a wealth of information about Saudis' knowledge, attitudes, and opinions about CTs. The respondents demonstrated a sufficient level of understanding of the benefits of CTs, what CTs entail, and the elements of informed consent. They expressed a conditional willingness to take part in a CT [3]. These findings, however, may not apply to other nations with diverse sociocultural backgrounds; as a result, it is critical to examine social, cultural, and economic views when doing research [10]. To our knowledge, there is no study on the knowledge and attitudes regarding clinical trial participation among the adult population in the Eastern Province of Saudi Arabia. Hence, this study aims to measure knowledge and attitudes and determine the factors that influence participation in clinical trials.

## Materials and methods

Study design

This cross-sectional study was conducted in the Eastern Province with 334 males and females. The study was conducted in the polyclinics center at King Faisal University, and the scientific committee of King Faisal University approved the study (KFU-REC-2022-AUG-ETHICS103). Participants included are Saudis living in the Eastern Province of Saudi Arabia, between 18 and 65 years old. Participants were excluded from the study if they were non-Saudi, lived outside the Eastern Province of Saudi Arabia, or were younger than 18 years and older than 65.

The target population was selected specifically for the Eastern Province because there is similar research conducted in another area of Saudi Arabia.

Data collection

Data was collected through a structured questionnaire formulated in Arabic and completed using Google Documents. The survey was distributed through Google Forms links online via social media platforms like WhatsApp (Meta Platforms, Inc., Menlo Park, California, United States) and Twitter (Twitter, Inc., San Francisco, California, United States). Consent was collected from the participants, which was the first question asked in the questionnaire, and the privacy of their information was ensured. The questionnaire was divided into four sections. Section 1 asked participants about their age, gender, educational level, marital status, and history of any chronic disease. Section 2 explored the respondents’ knowledge about the potential benefits of a CT, the essential elements of informed consent, and what a CT includes. Section 3 assessed participants’ attitudes concerning their willingness to participate in a CT, and Section 4 explored respondents’ perceptions towards participating in a CT. Adverbs of frequency scales are used in some of the questions.

Data analysis

The extracted data was revised, coded, and inputted into IBM SPSS Statistics for Windows, Version 22.0 (released 2013; IBM Corp., Armonk, New York, United States). All statistical analyses were completed using two-tailed tests. P values less than 0.05 were statistically significant. A descriptive analysis based on frequency and percent distribution was performed for all variables, including participants' personal data, sharing in CTs, and their perceptions. Also, data regarding participants' knowledge and attitudes toward CTs were tabulated and graphed.

## Results

A total of 334 participants completed the study questionnaire (Table 1). Participants' ages ranged from 18 to 65 years, with a mean age of 31.2 ± 13.9 years. Just over half of the sample was male (n = 189, 56.6%). Concerning the occupations of the sample, 141 (42.2%) were employed, 99 (29.6%) were students, and 77 (23.1%) were unemployed. Much of the sample held a university degree (n = 257, 76.9%) and were married (n = 202, 60.5% vs. n = 129, 38.6% unmarried). As for chronic diseases, 38 (11.4%) reported having hypertension, and 20 (6%) were diabetic. However, much of the sample (n = 269, 80.5%) reported they had no chronic health problems.

**Table 1:** Personal data of study participants, Eastern Region, Saudi Arabia (n=334) htn: hypertension; dm: diabetes mellitus

Personal data	Number	%
Age in years	-	-
18-29	143	42.8%
30-39	60	18.0%
40-49	77	23.1%
50+	54	16.2%
Gender	-	-
Male	189	56.6%
Female	145	43.4%
Work		
Not working	77	23.1%
Student	99	29.6%
Employed	141	42.2%
Private work	17	5.1%
Educational level	-	-
Secondary / below	61	18.3%
University graduated	257	76.9%
Post-graduate	16	4.8%
Marital status	-	-
Single	129	38.6%
Married	202	60.5%
Divorced / widow	3	.9%
Chronic diseases	-	-
None	269	80.5%
DM	20	6.0%
HTN	38	11.4%
Hypercholesterolemia	2	.6%
Asthma	6	1.8%
Renal diseases	5	1.5%
Cardiac disease	5	1.5%
Others	12	3.6%

Participants' awareness and perceptions of participating in a CT are presented in Table 2. Only 25 (7.5%) of the study participants were asked to join an RCT, of which 18 (72%) participated. A small portion of the sample (n = 39, 11.7%) knew someone who participated in a CT. When participants were asked about what comes to mind when hearing the term CT, 25.4% reported that CTs are used to evaluate a new drug, 15.3% reported that they are used to understand certain diseases, 4.8% believe that they are used to understand human behaviors, and 37.4% answered all were true. When asked who participates in these types of studies and for what purpose, 31.4% of participants reported sick volunteers are recruited for drug studies, 17.7% reported such studies are used to compare two drugs, 6% believed CTs recruited healthy volunteers, and 39.2% selected all the above.

**Table 2:** Participants awareness and perception of study participants, Eastern Region, Saudi Arabia (n=334)

Items	Number	%
Have you ever been asked to participate in RCT?	-	-
Yes	25	7.5%
No	309	92.5%
If yes, did you participate?	-	-
Yes	18	72.0%
No	7	28.0%
Do you know anyone who participated in RCT?	-	-
Yes	39	11.7%
No	295	88.3%
What comes to your mind when you hear the term clinical research?	-	-
Using new drug	85	25.4%
Understanding human behaviors	16	4.8%
Understanding certain disease	51	15.3%
Using new equipment	6	1.8%
Filling new questionnaire	9	2.7%
All of these	125	37.4%
I don't know	42	12.6%
What comes to your mind when you hear the term drug study?	-	-
A study conducted on healthy volunteers	20	6.0%
A study conducted on sick volunteers	105	31.4%
A study conducted to compare two drugs	59	17.7%
All of these	131	39.2%
I don't know	19	5.7%

In terms of the benefits of conducting CTs, most of the participants (86.5%) answered that participation in drug studies improves patient care (Table 3). Almost half of the sample (46.1%) agreed that CTs improve the welfare of society, and 7.5% did not believe there was any benefit.

**Table 3:** Participants' perception of RCTs sharing benefits (n=334) RCT: randomized controlled trials

Benefits	Yes		Maybe		No		I don't know	
	No	%	No	%	No	%	No	%
Improve patient care	289	86.5%	28	8.4%	9	2.7%	8	2.4%
Improve the welfare of society	154	46.1%	94	28.1%	64	19.2%	22	6.6%
No benefit	25	7.5%	35	10.5%	230	68.9%	44	13.2%

When asked about their attitudes toward participation in drug studies, 16% of the study participants agreed that participants in RCTs are subject to unnecessary risks (Table 4). Further, 29.6% reported feeling anxious about participating in a drug study, and 24.9% believe that their rights are being violated when a doctor asks them to participate in a drug study during a medical visit. A total of 41.3% think that participating in drug studies can be stressful for participants. On the other hand, 37.7% think that participants benefit from the studies, and 76.2% reported that drug studies may benefit those who develop the disease in the future. However, only 21.9% think that those who participate in drug studies receive the best treatment. Nevertheless, most participants agree that CTs are conducted in a responsible and ethical manner (59.9%), and 91% agreed that drug studies are necessary to develop any drug and determine its success.

**Table 4:** Participants attitude towards participation in drug studies, Eastern Region, Saudi Arabia (n=334)

Attitude	Number	%
Do participants take unnecessary risks?	-	-
Yes	51	16.0%
Maybe	126	39.5%
No	112	35.1%
I don't know	30	9.4%
Do participants benefit from these studies?	-	-
Yes	123	37.7%
Maybe	142	43.6%
No	36	11.0%
I don't know	25	7.7%
Do drug studies benefit those who develop the disease in the future?	-	-
Yes	246	76.2%
Maybe	55	17.0%
No	13	4.0%
I don't know	9	2.8%
How do you feel about participating in a drug study?	-	-
Anxious	99	29.6%
Unsure	117	35.0%
Comfortable	118	35.3%
Do you feel that your rights are being violated when your doctor asks you to participate in a drug study on the day of the medical visit?	-	-
Yes	83	24.9%
No	251	75.1%
Do you think that those who participate in drug studies receive the best treatment?	-	-
Yes	73	21.9%
Maybe	93	27.8%
No	59	17.7%
I don't know	109	32.6%
In general, do you think that participating in drug studies can be stressful for participants?	-	-
Yes	138	41.3%
Maybe	89	26.6%
No	46	13.8%
I don't know	61	18.3%
The best phrase that reflects your opinion about drug studies today?	-	-
The study is conducted in a responsible and ethical manner	200	59.9%
The study is conducted in an unethical manner	10	3.0%
The study is conducted by unqualified persons	8	2.4%
I have no opinion about drug studies	116	34.7%
Do you think that drug studies are necessary to develop any drug and determine its success?	-	-
Never	1	.3%
Rarely	6	1.8%
Sometimes	101	30.2%
Usually	203	60.8%
I don't know	23	6.9%

Concerning participation in drug studies, most participants (91.3%) reported they were more likely to participate if they understood the study well (Table 5). A large number of participants also felt that being able to obtain the results of the study after its completion (89.2%), the researcher having explained the study (85.9%), the personal physician having read the study protocol (84.1%), knowing the option to withdraw from the study at any time (80.2%), and having more time to think about it before agreeing (79.9%) were important factors. The least reported factor for participation was having a family member involved in the same study (58.4%).

**Table 5:** Factors affecting participants sharing in drug studies, Eastern Region, Saudi Arabia (n=334)

Factors	Yes		Maybe		No		I don't know	
	No	%	No	%	No	%	No	%
Participation of a family member in the same study	126	37.7%	69	20.7%	124	37.1%	15	4.5%
Possibility to consult your doctor about his opinion on participation	202	60.5%	52	15.6%	60	18.0%	20	6.0%
Possibility of having more time to think about it before agreeing	221	66.2%	46	13.8%	51	15.3%	16	4.8%
If the researchers are also willing to participate in the same study	208	62.3%	52	15.6%	52	15.6%	22	6.6%
The possibility of withdrawing from the study at any time	205	61.4%	63	18.9%	46	13.8%	20	6.0%
You understood the study well	264	79.0%	41	12.3%	19	5.7%	10	3.0%
Your personal physician has read the study protocol	224	67.1%	57	17.1%	40	12.0%	13	3.9%
The researcher has explained the study	230	68.9%	57	17.1%	38	11.4%	9	2.7%
Signing the declaration of participation in the study	189	56.6%	70	21.0%	57	17.1%	18	5.4%
Obtaining the results of the study after its completion	249	74.6%	49	14.7%	24	7.2%	12	3.6%

When asked about their reasons for considering participation in drug studies, the most reported reason was helping the community and others (92.8%; Table 6), followed by assisting in the obtainability of new drugs (90.4%), contributing to scientific knowledge (89.8%), and receiving the best medical care. As for barriers, the most mentioned were lack of knowledge of drug studies (84.1%), fear of risk (76.9%), and lack of time (64.7%).

**Table 6:** Reasons and barriers of participation in drug studies, Eastern Region, Saudi Arabia (n=334)

Reasons and barriers	Yes		Maybe		No		I don't know	
	No	%	No	%	No	%	No	%
Reasons for participation in drug studies	-	-	-	-	-	-	-	-
Receive the best medical care	234	70.1%	64	19.2%	22	6.6%	14	4.2%
Contribute to scientific knowledge	250	74.9%	50	15.0%	22	6.6%	12	3.6%
Helping the community and others	273	81.7%	37	11.1%	10	3.0%	14	4.2%
Obtaining financial compensation	102	30.5%	80	24.0%	128	38.3%	24	7.2%
Assisting in the obtainability of new drugs	247	74.0%	55	16.5%	18	5.4%	14	4.2%
Feeling obligated (embarrassed) towards the person (researcher) asking	98	29.3%	86	25.7%	128	38.3%	22	6.6%
Barriers to participation in drug studies	-	-	-	-	-	-	-	-
Fear of risk sharing	200	59.9%	57	17.1%	60	18.0%	17	5.1%
Lack of time	126	37.7%	90	26.9%	102	30.5%	16	4.8%
Lack of knowledge in drug studies	205	61.4%	76	22.8%	40	12.0%	13	3.9%
Health-related conditions	129	38.6%	75	22.5%	109	32.6%	21	6.3%
Lack of confidence in the medical system	97	29.0%	81	24.3%	138	41.3%	18	5.4%
Lack of financial compensation	107	32.0%	61	18.3%	140	41.9%	26	7.8%

**Figure 1:**
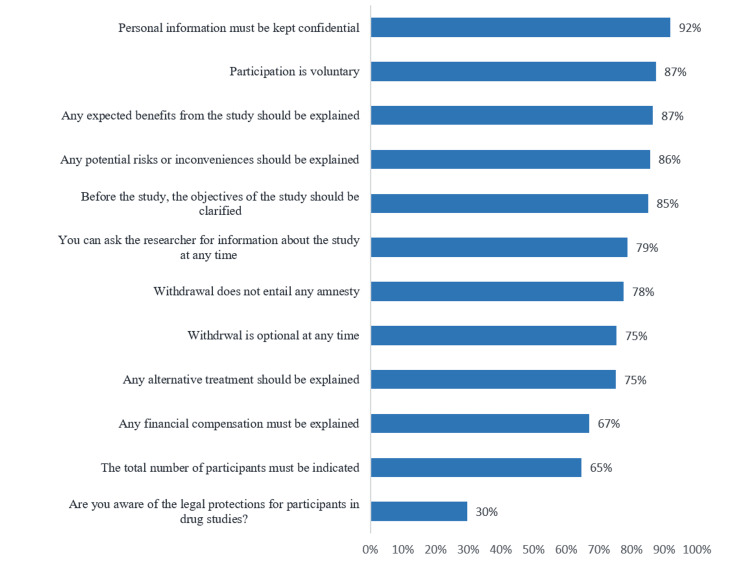
Participants' knowledge of clinical trial participation, Eastern Region, Saudi Arabia

We also asked participants about their knowledge concerning CT participation. Almost all the participants reported that they understood that personal information must be kept confidential (92%; Figure 1). Most also understood that participation is voluntary (87%), any expected benefits from the study should be explained (87%), any potential risks or inconveniences should be explained (86%), and before the study, the objectives of the study should be clarified (85%). Fewer participants reported that they would want to know the total number of participants (65%) and the legal protections for participants in drug studies (30%).

Participants, when asked how likely they were to participate in CTs, said that around 74.9% would participate, especially if they were in excellent health, with the intention of helping others. In addition, 63.2% will participate even if they have a severe illness and 60.2% if they have a slight illness (Table 7).

**Table 7:** Participants' practice regarding clinical trial participation, Eastern Region, Saudi Arabia (n=334)

Practice	Yes		Maybe		No		I do not know	
	No	%	No	%	No	%	No	%
If you have a minor illness	111	33.2%	90	26.9%	105	31.4%	28	8.4%
If you have a severe illness	150	44.9%	61	18.3%	92	27.5%	31	9.3%
If you are in good health but share with the aim of helping others	169	50.6%	81	24.3%	59	17.7%	25	7.5%
Will never participate	56	16.8%	77	23.1%	141	42.2%	60	18.0%

## Discussion

CTs are key milestones in modern medicine for developing and identifying successful medicines [11]. However, evidence shows that, in comparison to other areas and nations, the number of clinical research studies undertaken in the Middle East is comparatively modest and falling behind. Specifically, only 8,706 of the 211,437 registered clinical trials worldwide in 2015 were undertaken in the Middle East, with only 409 completed in Saudi Arabia [5]. In 2019, a systematic review published in the Saudi Pharmaceutical Journal reviewed the status of clinical trials in Saudi Arabia and identified several factors that may be contributing to the low number of trials, including a lack of trained and experienced investigators, limited funding and infrastructure, and regulatory barriers [12]. We hypothesized that one of the reasons for the lack of CTs in Saudi Arabia is due to poor response rates among the population. Thus, identifying and comprehending the barriers that prevent patients from enrolling in CTs is essential for increasing patient involvement. To the best of our knowledge, there is limited information about the factors that drive CT involvement in Saudi Arabia. Therefore, our study is one of the first to focus on the knowledge, attitudes, and perceptions of Saudis toward CTs.

Overall, our findings demonstrate a general lack of understanding of CTs. Although most participants were aware that their participation in CTs was optional, most were unaware of their right to withdraw from CTs and the legal protections of participants in drug studies. Similar findings in research conducted in healthcare settings (with patients and/or their families) within the KSA support the current findings [5]. One reason for the lack of knowledge among Saudis could be due to a lack of institutional and national CT promotion activities [13].

Our findings suggest that addressing the factors that prevent people from enrolling in drug studies and spreading awareness about the importance of these studies is critical for increasing the number of participants. Participants in our study reported that they believed CTs were important for improving patient care, developing new medications, and finding which diagnostic test worked best. However, regardless of the benefits they identified, participants noted several barriers to participating in CTs, especially a lack of knowledge of drug studies (84.1%). Thus, by improving the level of knowledge in society and emphasizing the importance of CTs and how their participation can help in creating future treatments, we will be able to increase the number of participants.

In our study, respondents expressed a conditional willingness to participate in a CT. We also discovered that the subject of trust was an essential factor for people invited to take part in a CT. Since physicians are frequently key sources of information for patients making decisions about CT participation, over 60.5% of respondents stated that they would need to talk with their physician before participating in a CT. Similarly, Tanai et al. discovered that CT design and the doctor-patient interaction might have a significant influence on patient engagement in a CT scan [11].

Our research also revealed that participants in a CT would feel more comfortable engaging in a study if the study protocol was clear to them, they read it, and the researcher fully described the study's methodology. Most respondents (81.7%) also reported assisting society as a motive for taking part in a CT. As a result, it is critical that investigators evaluate these perspectives and work to improve patients' knowledge of the specific research topic and how their participation will benefit society. According to Fallowfield et al., offering more information enhances a person's desire to enroll in a CT [14]. Other studies, however, have found that even when research personnel clarify topics, study participants may not grasp them [15].

While interpreting the results of this study, a few limitations should be taken into consideration, including the small sample size and the distribution of the self-administered questionnaire that was distributed online through social media platforms, which affect the generalizability of the results. Also, the study was conducted in one region of Saudi Arabia. Therefore, the results may not be representative of the rest of the country.

## Conclusions

The purpose of this study was to evaluate the knowledge and attitudes of the adult population in the Eastern Province towards participating in clinical trials (CTs). This study has identified that the majority of the participants are not aware of CTs. Increasing patient knowledge about CTs is essential. To increase knowledge and CT recruitment and guarantee equal gains from scientific research and discovery, multimodal strategies (e.g., increased patient-provider communication and online information for sharing trial information) may be successful. Based on our results, physicians and healthcare providers in the Eastern Province are in the best position to encourage patients to participate in CTs. However, patients are only likely to participate if they understand the study and any expected benefits and risks from their participation. Future research should consider the barriers and prescribed factors for interested doctors to perform CTs.
